# New Device for Epistaxis

**Published:** 1875-11

**Authors:** 


### Editorial.

---

New Device for Epistaxis.—Dr. S. G. Riley, of Shiloh, Ga., sends us the following:

"A death having recently occurred in this county from epistaxis, I am stimulated to send you the suggestion of a remedy, which is obtainable at every farm house, and which I have not in any instance known to fail in promptly arresting epistaxis. The following is the method:

"From the entrails of a recently killed chicken, select a gut of suitable size to properly fill the nasal fossa when distended, and having cleansed it properly, secure one end well with a ligature, and then slip the gut over the nozzle of a small syringe, previously filled with cold water, in such manner as to carry the point of the syringe down to the *cul-de-sac* found by the ligated gut, the latter being plaited upon the syringe pipe, and secure the other end of the gut upon the pipe, water tight, by another ligature, and the apparatus is ready for use. The armed pipe is now passed into the nostril, and the piston of the syringe slowly pressed into the barrel. As the water escapes the distended gut *worms its way up the nasal passage*, insinuating itself into every crevice, and producing an equable compression everywhere, which may be increased in force until the bleeding points are fully and effectually compressed. Whilst the water is being thrown in the pressure induced in the nostril will cause the syringe pipe to retreat from the nasal cavity, so that a ligature may be applied to the distended gut at a point just external to the nostril, and the syringe removed, whilst the hemostatic pressure is maintained as long as may be desired.

"It is proper that I should say that this device is not original with myself, as I learned it from an old lady in Texas, who made the suggestion, with happy result, in a case which had defied all other remedies and appliances. I have since used it in a number of obstinate cases, and always with success."

